# Supplementary material for: Dynamic transcriptomic profiles of zebrafish gills in response to zinc supplementation
Source: BMC Genomics. 2010 Oct 11;11:553. doi: 10.1186/1471-2164-11-553 (PMC3091702; doi:10.1186/1471-2164-11-553)
Supplement: Additional file 2 — Interactive Direct Interaction Network representing the molecular interactions between zinc, copper, iron, calcium and proteins encoded by transcripts changed by zinc supplementation. Mini web-site containing index.html and hyperlinked pages in subdirectory describing a Direct Interaction Network automatically generated based on curated interactions contained within the proprietary PathwayArchitect database. Ovals represent proteins and the circles symbolize metal ions. Objects are coloured by their abundance in zebrafish at the time-point they were significantly different from the control is a scale from -4 fold (dark green) to +4 fold (dark red). Where significant differences were found at more than one time-point, the colour overlay shows expression at the first instance. Dark blue squares denote 'binding', and light blue squares 'expression'; green squares stand for 'regulation', green diamonds for 'metabolism', and green circles for 'promoter binding'. Arrow heads indicate directionality of the interaction where annotated. All nodes and edges can be further interrogated by selecting the relative area of the image. [file 1471-2164-11-553-S2.zip › PathwayArchitect Zn xs DIN/427829.html]

# REGULATION:

|  |  |
| --- | --- |
| Type | REGULATION |
| Effect | Positive |


---

|  |  |
| --- | --- |
| Score | 0 |


---

|  |  |
| --- | --- |
| Reference Count | 5 |


---

|  |  |
| --- | --- |
| Mechanism | Unknown |


---

|  |  |
| --- | --- |
| Reference:0 || Sentence | "CONCLUSIONS: These results suggest that propofol increases myofilament Ca2+ sensitivity via a PKC-dependent pathway involving the phosphorylation of MLC2." |
| PMID | 12766644 |
| Year | 2003 |
| Species | Rat |
| Journal | Anesthesiology |
| RefScore | 1 |
| Source | PArchNLP |
  |
|  |  |


---

|  |  |
| --- | --- |
 Reference:1 || Sentence | "In addition, phosphorylation of MLC2 in myofibrils by MLCK increased both the Ca(2+)-sensitivity and maximum activity of the myofibrillar Ca(2+)-stimulated MgATPase activity." |
| PMID | 8503915 |
| Year | 1993 |
| Species | Rat |
| Journal | Biochem Biophys Res Commun |
| RefScore | 1 |
| Source | PArchNLP |
  ||  |  |


---

|  |  |
| --- | --- |
 Reference:2 || Sentence | "Regulation of Ca(2+)-sensitivity via MLC-2 phosphorylation may be a potential compensatory mechanism to reverse the detrimental effects of increased Ca(2+)-sensitivity and impaired Ca(2+)-handling on diastolic function in human heart failure." |
| PMID | 12566123 |
| Year | 2003 |
| Species | Human |
| Journal | Cardiovasc Res |
| RefScore | 1 |
| Source | PArchNLP |
  ||  |  |


---

|  |  |
| --- | --- |
 Reference:3 || Sentence | "The present results show that that in rat mesenteric small arteries noradrenaline-induced Ca2+ sensitization is associated with an increased proportion of phosphorylated MLC." |
| PMID | 9706005 |
| Year | 1998 |
| Species | Rat |
| Journal | J Physiol |
| RefScore | 0 |
| Source | PArchNLP |
  ||  |  |


---

|  |  |
| --- | --- |
 Reference:4 || Sentence | "Treatment with ATP gamma S in the presence of the MLC kinase inhibitor ML-9 increased the sensitivity to Ca2+ and abolished the response to noradrenaline. 4." |
| PMID | 9706005 |
| Year | 1998 |
| Species | Rat |
| Journal | J Physiol |
| RefScore | 3 |
| Source | PArchNLP |
  |


---

|  |  |
| --- | --- |
